# The Yellow Fever Bacillus

**Published:** 1897-10

**Authors:** 


### The Yellow Fever Bacillus.

From a morphologicol point of view, says M. J. Sanarelli in the *Semaine Medicale* for July 7th (*Independence Medicale*, July 28th), this bacillus is like a small stick with rounded ends; it is found in pairs in cultures and in small groups in the tissues; it varies in length, and is generally two or three times as long as it is wide; it is rather polymorphous.

The author states that searching for it in the tissues gives unsatisfactory results, except in cases in which the death of the patient occurs without secondary septicæmia. Also in cases in which the bacteriological result is the most distinct it is not easy to ascertain the presence of the icteroid bacilli on the sections of tissue because of their number being often restricted. Nevertheless, it is possible to find these microbes in the organs more frequently united in small groups, and located always in the small capillaries of the liver, the kidneys, etc.

The best means for demonstrating not only the presence of the icteroid bacillus, but also its tendency to localize in small groups, especially in the capillaries, consists in taking a piece of the liver of a person recently dead and keeping it in a steam bath at a temperature of 58.3° F. for twelve hours, so as to facilitate the multiplication of the specific microbe.

The bacillus of yellow fever, says M. Sanorelli, is rather easily developed in all the ordinary nutritive media. In cultures in patches and on ordinary gelatin it forms round, transparent and granular colonies which present during the first three or four days the appearance of leucocytes; later, the granulation of the colony becomes more intense, and ordinarily, a central or peripheral nucleus, completely opaque, is formed; after a time the colony itself becomes altogether opaque and never liquefies the gelatin. The striated cultures on gelatin, which are indirectly solidified, are developed in the form of brilliant and opaque drops similar to drops of milk. In bouillon the icteroid bacillus is developed slightly without forming films or flaky deposits. In solidified blood serum it grows in an almost imperceptible manner. The cultures on gelose, on the contrary, differ from those which occur in the case of the majority of the known pathogenic microbes, and represent for the icteroid bacillus a diagnostic measure of the first order, but only in certain conditions.

When the colonies are developed in the steam bath their appearance does not difier from that of a number of other microbian species; they are round, of a grayish color, somewhat iridescent, and transparent, with a smooth surface and regular borders. If, instead of developing them in a steam bath at a temperature of 98.3°, they are allowed to grow at a temperature of from 71.3° to 78.4° F., the colonies will have the appearance of drops of milk, opaque, prominent, with a pearly reflection, and completely different from those developed in the steam bath.

This difference in the development, says the author, may be made use of by subjecting the cultures for twelve or sixteen hours first to the steam bath, and afterward to the ordinary temperature for the same length of time. The colonies will then be seen to consist of a flattened central nucleus, transparent and bluish in color, surrounded by a prominent and opaque peripheral circle, and presenting, as a whole, the appearance of a wax seal. This characteristic, M. Sanarelli thinks, should be considered, for the present, at least, as specific, and, as it is developed in less than twenty-four hours, it serves to establish very rapidly and surely the bacteriological determination of the icteroid bacillus. Aside from this morphological characteristic, which enables us to distinguish the microbe of yellow fever from all others which are known, the icteroid bacillus is endowed with some interesting biological properties.

It is an ectogenous anærobion, and does not resist Gram's coloring; it imperceptibly causes the fermentation of lactose and more actively that of glucose and saccarose, bnt it is incapable of coagulating milk; it is very resistant to desiccation, dies in water at 140° F., and is killed by the sun's rays in seven hours; it lives a long time in sea water.

Tha specific microbe of yellow fever, M. Saranelli states, is pathogenic in the majority of domestic animals; few microbes have a pathological domain that is so varied and extended. The steatogenic properties are manifested with a much greater intensity if the animal experimented upon occupies a high rank in the zoological world.

The congestive and hemorrhagic properties, while being common to various kinds of virus, constitute, through the anatomical location where they preferably exert their influence, a very marked specific characteristic. It is to them, in fact, that not only the classic vomiting of blood and the various hemorrhagic manifestations are due, but also the congestion of the blood-vessels, which is the principal cause of the pathognomonic pains in yellow fever.

Its emetic properties, while they are not so strictly specific as those previously mentioned, give to this virus, by the rapidity, the intensity, and the persistence with which they are manifested in man and the higher animals, a pathogenic characteristic which enables us to distinguish it easily from all the other viruses known at the present time.

The comparative rarity with which the icteroid bacillus is found in the human organism and the violence of the symptoms which may be provoked in the dog soon after the intravenous injection of a comparatively abundant culture would lead to the supposition of the existence of a very active specific poison. The author studied this poison, which was obtained by simply filtering a culture of the icteroid bacillus which had been in bouillon from five to twenty-five days.

The yellow-fever poison tolerated, almost with impunity, steaming at 152° F., but the temperature of boiling water perceptibly weakened it. The author also studied the action of this specific poison on the guinea-pig, the rabbit, the dog, the cat, the goat, the donkey, the horse, and man.

The experiments on the human subject were five in number. The injection of a filtered culture, in a comparatively weak dose, produced in man typical yellow fever accompanied by its anatomical and symptomatic train. This fact, the author thinks, not only is a convincing proof of the specific value of the icteroid bacillus, but establishes on new lines the ætiological and pathogenic conception of yellow fever, and shows that all of these symptoms are due only to the poison produced by the microbe circulating in the blood.

All the symptomatic phenomena, all the functional alterations, and all the anatomical lesions of yellow fever, he says, are the result only of the steatogenic, the emetic, and the hæmatolytic action of the toxic substance produced by the icteroid bacillus. —*New York Medical Journal.*

---

**One** should not expectorate on open street-cars and expect to rate as a gentleman.—*T. C. M., Lancet-Clinic.*

---

**During** the administration of ether the most alarming danger signals are sudden pallor of the face, dilatation of the pupils, and darkening of the blood. When the symptoms present themselves, the anæsthetic should at once be withdrawn, and resuscitating measures instituted.—*Hearn.*

---

**Of the** original members of the American Medical Association only four of those present at its organization are still living. These are Dr. Nathan S. Davis, of Chicago, an ex-president; Dr. John B. Johnson, of St. Louis, an ex-vice-president; Dr. Alfred Stille, of Philadelphia, an ex-president, and Dr. David F. Atwalter, of Springfield, Mass. These constitute the revered remnant of a once able and aggressive body of medical men whose purpose was to lay broadly and enduringly the foundation of medical science on American soil. They builded even better than they knew, and the present generation is in no wise tardy in a generous expression of its application of their noble and successful work.—*Ex.*

